# Supplementary figures and images for: Validation of a deep learning computer aided system for CT based lung nodule detection, classification, and growth rate estimation in a routine clinical population
Source: PLoS One. 2022 May 5;17(5):e0266799. doi: 10.1371/journal.pone.0266799 (PMC9070877; doi:10.1371/journal.pone.0266799)

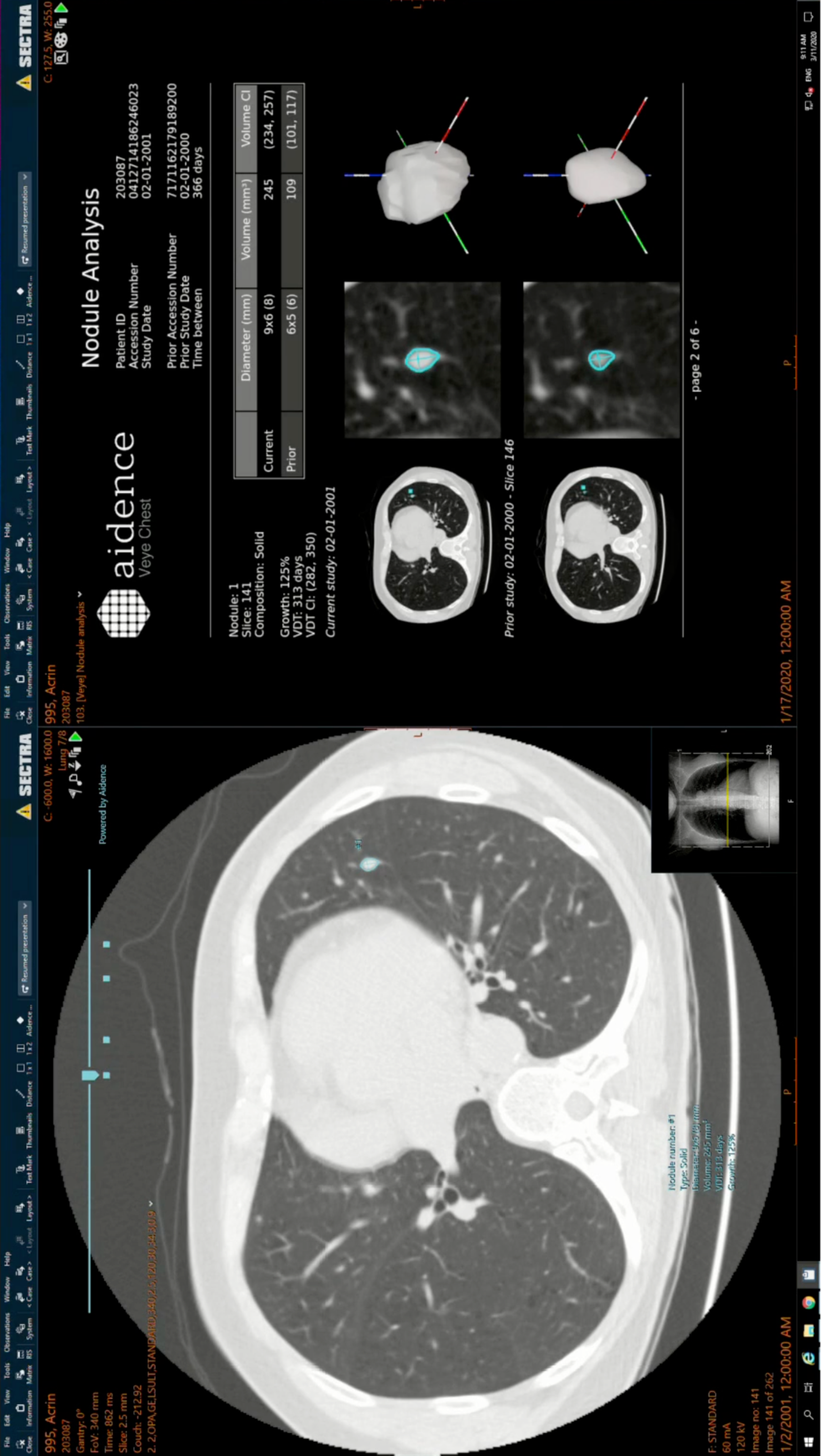

Supplement: S1 Fig — (TIF) [file pone.0266799.s001.tif]
