# Supplementary material for: Validation of a deep learning computer aided system for CT based lung nodule detection, classification, and growth rate estimation in a routine clinical population
Source: PLoS One. 2022 May 5;17(5):e0266799. doi: 10.1371/journal.pone.0266799 (PMC9070877; doi:10.1371/journal.pone.0266799)
Supplement: Attachment — Submitted filename: Response to Reviewers.docx [file pone.0266799.s002.docx]

# **Response to reviewers**

Dear PLOS editorial team, reviewer #1 and reviewer #2,

Thank you for your comments, critical questions and suggestions. We have updated the manuscript accordingly and have addressed your comments in the response letter below.

Our apologies for our late reply. The reason we give for this is that the software analyzed in this study is developed by a start-up company with limited resources. Most of our efforts in the past months have gone to additional studies that are required to obtain 510(k) approval from the FDA.

Our CAD has been further developed since the study described in this paper and has increased in performance, both in sensitivity and specificity. Yet, we believe it is still valuable for the scientific literature to publish our initial clinical validation of the software. We believe that transparency is one of the most fundamental values in science.

Kind Regards,

Jeroen Nijwening

# **Response to Academic editors**

Dear academic editors, we have adapted the style of the manuscript as requested. Furthermore, we have included a new cover letter which contains the updated information regarding the funding statement and competing interest statement.

# **Response to Reviewer #1**

1. The important points are well organized in the abstract.

Thank you for your compliment regarding the abstract. We have revised the abstract based on the reviewers’ suggestions and expect that it still holds the core message of the manuscript.

2. Introduction: It reflected well the reality and difficulties of detecting pulmonary nodules on CT scans. In addition, the authors well explained the importance of CAD regarding this point.

Thank you for highlighting the importance of CAD within a radiologist’s practice. We believe that CAD will improve the quality of reports and will decrease the amount of time spent on decision making.

3. Subject selection - exclusion criteria: the authors excluded diffuse pulmonary disease such as ILD. What threshold they used for this exclusion? If patients had very subtle ILD (or ILA), were they excluded? And how could the authors know the existence of ILD? They reviewed all the CT exams? Please clarify.

All studies were initially selected based on the reports in the electronic health records. This provided us with a clinical diagnosis, including the presence of lung nodules or other concomitant lung diseases. If the diagnosis of a diffuse pulmonary disease, like ILD, was made in the clinical report, these studies were excluded. However, emphysema, likely from a smoking history, was We have clarified this in the materials and methods section, lines 121 to 123.

4. (There was no comment #4)

5. Subject selection- group categorization seems cumbersome for future readers and the authors described that Group 1 consisted of 178 CT scans reported as being free from pulmonary nodules. But the result (Table 1) is different. It was read that there was no pulmonary nodule, but were these cases actually had pulmonary nodules?

We thank the reviewer for this excellent question and observation. We selected CT studies based on the clinical report, but (almost as expected), some lung nodules had been missed and therefore were only reported during this study. As these were historical cases, we did not evaluate whether this could have had an impact on the patient outcome, given that malignancy should have become clear before the start of this selection process. We have clarified this in the materials and methods section, line 127 and 128 and in the results, lines 244 and 245.

6. Only one CT machine was used in this study. If so, it seems very unified protocols and well organized study protocol. If not, please state more clear about types of CT machines.

This was a single center study, using historical cases. We selected a period of stability, and therefore the work was done on a single vendor system. We appreciate that this may be a weakness, and have highlighted this in the discussion. We have addressed this in the discussion section, lines 331 to 338.

7. Nodule definition - please consider to delete "“pulmonary nodule” was not firmly defined since the notion of nodule may not represent a single entity capable of verbal definition." It seems unnecessary.

We thank the reviewer for this suggestion and have removed this sentence.

8. Nodule definition - the authors defined "actionable nodules" as a largest axial diameter between ≥5mm (or a volume of ≥80mm3) and ≤30mm. I wonder whether they have some references for this definition or they arbitrarily decided it.

“Actionable nodules” means nodules that require follow-up based on risk of malignancy. We have applied the British Thoracic Society guidelines on nodule management (reference: Callister *et al*., 2015), and have added this to the end of the sentence.

9. CAD software - For readers who are not friendly with this commercially available CAD system, please add detailed information about the CAD. For example, 1) why the threshold was decided as 0.1? what does it mean? 2) which CT examinations were used for its development in terms of slice thickness or use of contrast media?

Thank you for suggesting to add this information. Based on this comment and others comments from reviewer #1 and #2 we have adapted the chapter about the CAD software.

In brief: the operating point was configured to yield a high sensitivity in order to detect as many actionable nodules as possible. Configuration favoring sensitivity automatically also yields a high number of false positives.

The device was developed using a lung cancer screening cohort using appropriate screening scanning protocols which include slice thickness <=3mm and without contrast media. A part for conducting the study described in the manuscript was to investigate how the AI models (which were trained on screening scans) would relate to scans obtained in routine clinical practice.

10. Image annotation - "three different groups: micro-nodules, masses, benign nodules and non-nodules." Not three different groups. Please revise it.

Thank you for this remark, we will change the text to: “four different groups”.

11. Reference standard and Data analysis -The description is rather complex and difficult to understand. I hope it is organized so that it is easy to understand.

We thank both reviewers for this comment. We have tried to clarify the process in the materials and methods section. The nodules were read independently, and blinded for clinical information, by two experienced chest radiologists, with a third senior chest radiologist available if no consensus was reached.

12. Reference standard and Data analysis -The most curious thing is that the authors created a reference standard as a result of the panels consisting of readers 1, 2, and 3. Nevertheless, it seems that the performance evaluation of readers was done as this reference standard.

The readers have read each case unaided and aided by the CAD (concurrent reader). As there were independent reads in place, we were able to construct a consensus report for all nodules, involved a third reader where necessary, which served as the reference standard.

13. Results: The content of the result may seem appropriate, but revision should be made according to the above items being modified.

We thank the reviewer for his/her helpful comments, and hope that the changes made according to the suggestion have clarified questions and improved the manuscript accordingly.

# **Response to Reviewer #2**

We thank reviewer #2 for understanding the message of our manuscript by providing a correct and concise summary of the research and its objectives.

Major comments of reviewer #2:

1.  The CAD system that is under investigation here is used for setting the reference standard. In addition, the readers for which the aided and unaided performance is reported, are used to make the reference standard. This affects the results and potentially positively biases the CAD results. The authors have also mentioned this in the Discussion so it is recognized as a limitation already. I think the conclusions of this study should therefore be less strong. It would be best if two other readers would also split all cases and read all of them without CAD support. Then, a truly independent read would be available. Ideally, this reader would read with and without CAD, but then, all cases need to be read twice, so is more effort.

We thank the reviewer for this comment. We wish to make it clear that the reference standard was created by a consensus report of up to three (where required) experienced chest radiologists. The software was indeed part of creating the reference” standard, but we opted to test the software by allowing readers to see 50% of cases with added software information to assess its impact.

2. Related to the first point: A proper experiment setup for an observer study to compare aided vs unaided reads of data is multi-reader multi-case (MRMC) analysis, which also allows for better statistical comparisons than the t-tests that are performed now. If it is still possible to ask additional readers, that would make the study stronger. In addition, please consult with a statistician to see whether a form of MRMC analysis is possible on this data.

As stated under point 1, we only investigated the potential impact of the software tool once the readers had independently read the studies and reached consensus. We apologize this wasn’t made clear, and have amended the text.

3. The CAD is used at a setting with high sensitivity and relatively high false positive rates. If this is the setting that the radiologists used while reading the cases, then the paper should also report the standalone performance of the CAD system at this operating point. It is not clear to me what the performance is at this operating point. Several operating points are reported by the authors, but it is not clear to me which one correspond to the setting used by the readers (threshold of 0.1). Please add that as a dot on the FROC curve in Figure 1. Please also discuss what effect this setting may have on the study results in the Discussion part.

We thank the reviewer for this comment and have added this to the text, see lines 284 – 286. The standalone performance of the CAD, when set to 0.1, correlates to an average sensitivity of 95% and an average number of 7 false positives per study based on this dataset.

4. Is the current CAD system approved/cleared for clinical use as a second reader or concurrent reader? This is not clear to me.

The current CAD system is CE certified as a second **and** concurrent reader.

5. Some important related literature in this area is not covered, for example: <https://www.ajronline.org/doi/pdf/10.2214/AJR.17.18718>

We thank the reviewer for this suggestion, and have incorporated this reference to the introduction and discussion.

6. A table which gives a breakdown of the CAD marks and the TP and FP categories would be very useful. The authors wrote "Finally, the readers classified all FP CAD prompts into three different groups: micro-nodules (largest axial diameter <3mm), masses (largest axial diameter >30mm), benign nodules (benign calcification pattern or clear benign perifissural appearance) and non-nodules (pleural plaque, scar tissue, atelectasis, fibrosis, fissure thickening, pleural fluid, pleural thickening, intrapulmonary vessels, consolidations, outside of lung tissue, or other (free format))." So, for every CAD mark, this rating is available so would be great to see a table with this information.

We agree that this is useful information to add to the manuscript, thank you for the suggestion. Instead of adding an extra table to the paper, we have added this information to the text in the M&M section on line 178 to 180.

Detailed comments of reviewer #2:

7. Please be more clear about the selection of the CT cases. If I understand correctly, the original radiologist reports were manually checked to see whether nodules were reported or not, and that gave groups 1 and 2. But then the abstract should not state the exact nodule numbers yet, because I suppose this is the result after the annotation process, so should be in the Results section of the abstract.

Thank you for this suggestion, we have changed the abstract accordingly. We acknowledge that the total number of cases per group are part of the result section.

8. The statement on Data Availability: I understand that the scans cannot be shared, but it would be good if the result files with the readings can be shared. So, a csv file with for each cases the recorded nodules per reader, and whether they were in the end part of the reference standard, etc.

We have made a csv file with all raw data available and have uploaded this to the PLOS One site.

9. How many CAD marks in total were there at the threshold of 0.1?

The standalone performance at a detection threshold of 0.1 are: 327 actionable nodules – 16 false negatives (FN) = 311 true positives (TP); 311 TP + 1927 false positives (FP) = 2238 CAD marks.

10. How many CAD marks were there on micronodules or masses or marks only annotated by one reader and thus ignored in the FROC analysis? Please report.

Since the inclusion criteria were: all nodules ≥ 5 mm / ≥ 80 mm^3^ AND < 30 mm based on average segmentation. The following nodules were excluded based on the above criteria: nodules without majority consensus: 208; nodules < 5mm or < 80 mm^3^: 86; masses (≥ 30 mm): 6; benign nodules (calcified or perifissural nodules with actionable size: 59.
