# Supplementary material for: Validation of a deep learning computer aided system for CT based lung nodule detection, classification, and growth rate estimation in a routine clinical population
Source: PLoS One. 2022 May 5;17(5):e0266799. doi: 10.1371/journal.pone.0266799 (PMC9070877; doi:10.1371/journal.pone.0266799)
Supplement: Attachment — Submitted filename: Response_to_Reviewers.docx [file pone.0266799.s003.docx]

## Rebuttal letter, second response to reviewers

**Reviewer #1:** Thank you for submitting the manuscript. I raised the following queries to improve the quality of this study

1. Page 16, lines 95-96: Please specify the diagnostic performance of radiologists (sub-specialist or specialist) for future readers to be interested in this manuscript and compare the performance with that of the CAD’s system.

As you suggested, we have added the corresponding performance from the cited papers to the text. However, we cannot compare this performance to what we find in our study (with and without CAD support) because the introduction is not the place to add this. We do compare our results with that of other studies in the Discussion section.

2. Page 16, line 101-102: Please clarify how different malignancy probability pulmonary nodules have as they are solid or subsolid nodules presented in the CT examinations.

We thank the reviewer for this observation and have added to the manuscript that sub-solid nodules are likely to be malignant (see Track Changes). In the current guidelines there is not yet a clear cut off or probability factor for malignancy. Sub-solid nodules are more often identified as malignant when analyzed (after a biopsy / resection, or via PET). The Fleischner recommendation in the cited paper states the following about sub-solid nodules: “Solitary part-solid GGNs, especially those in which the solid component is larger than 5 mm, should be considered malignant until proved otherwise provided either growth or no change is seen at a follow-up CT examination performed in 3 months”.

3. Page 18, line 117: Please give specific intervals the subjects participants in this study instead of “obtained at least 5 years prior to this study.”

Thank you for this remark. Based on this and your following comment, we have rewritten the whole “Subject Selection” part to be more clear about the procedure of collecting the scans for the study, see the rewritten part in Track Changes. In addition, we would like to add that CT studies between January 2008 and December 2009 were searched for potential inclusion into this study. This gave us a follow-up period of more than 7 years, to enable potential reference standard to be assessed.

4. The “subject selection” section is somewhat complicated. The authors should suggest a flowchart for selecting the study population as Figure.

This is a good suggestion. We realize that the “Subject Selection” paragraph could be confusing and we would like to suggest to rewrite this paragraph in a concise way instead of adding a flowchart as an extra figure to the text. We hope that the reviewer and editor agree to this suggestion. Please find the rewritten part in the uploaded “Revised Manuscript with Track Changes”.

5. As can be seen from the title, this study is a validation study in the routine clinical population. In fact, there are cases where there are more than 10 pulmonary nodules in routine clinical practice, and there is no other mention of this. Please explain this.

This is a good question and we understand why the reviewer is asking this since, indeed, we see cases with more than 10 nodules in clinical practice. For this study, however, we choose for simplicity reasons to cap the maximum amount of nodules in a study to 10. Since in our population the vast majority of patients has less than 10 nodules. Adding studies of more than 10 nodules per patient would not add more value to the quality of the study.

6. Please suggest all CT scanners included in this study.

These are the number of studies performed with a specific CT scanner:

- Toshiba 333 Aquillion: 330
- Toshiba Aquillion-CX: 2
- Toshiba Aquillion ONE: 1
- GE Medical Systems LightSpeed 16: 2
- GE Medical Systems LightSpeed: 2

We have added this to the revised manuscript.

7. There is no figure using the Veye Chest software in this study. Since most future readers are unfamiliar with this software, the authors will need a representative figure of how it works.

We would like to thank the reviewer for this excellent suggestion. We have added a screenshot of the actual product to the supplementary material. I do want to stress, however, that the intention of this manuscript is non-promotional and therefore we want to exclude branded material as much as possible from the main body of text and figures.

8. Why did the authors set the threshold of the software as 0.1? Of course, sensitivity is vital importance in the screening setting, the authors should perform various settings with various threshold values (for example, 0.1, 0.3, 0.5, 0.7) to adjust false-positive results.

We would like to thank the reviewer for this question and expect that the following answer suffices. As discussed in the first paragraph in the Discussion, the threshold of 0.1 was based on a sensitivity of 95% and a false-positive rate of 7, which is a workable number of false-positives. We have done a variation of the thresholds, as demonstrated in the range of threshold of false positive lung nodules in the results section. In addition, the nodule software was not used as a screening tool, rather for evaluation of incidental pulmonary nodules in a routine chest CT population using standard-dose CT protocol. In this setting, it is commonplace to evaluate at a threshold of 80 mm^3^ or 5 mm detection level, and for this threshold, the software setting of 0.1 is the optimal setting.

9. “The detection results of CAD were made available at random in half the scans.” conflict to the following sentences. The CAD system was not all CT scans? Please revise this part appropriately.

Thank you for this remark. We realize the set-up of our study could be more clarified and have adjusted the manuscript accordingly. Briefly, the CT-studies were divided in two parts, A and B. Reader 1 analyzed the studies in part A with CAD and part B without CAD. For Reader 2 this was the other way around: studies in part A were analyzed without CAD and part B with CAD. Discrepancies were compared. Reader 3 subsequently adjudicated all discrepancies without the results of CAD.

10. Page 20, line 168-169: Please add the reference of the “Any nodules requiring follow-up according to lung cancer screening criteria were classified as “actionable nodules.”

Thank you for this suggestion, we have added reference 10 behind this sentence because we are referring to the same reference as is cited in the Nodule Definition paragraph in the Materials and Methods section.

11. Why the readers evaluated the CT features (solid or sub-solid) on a tablet? I believe that a dedicated workstation for reading CT scans is appropriate.

Good question from the reviewer, we would like to give the following explanation: “We used a high-definition iPAD Pro tablet in order to facilitate this study, which was performed by three different radiologists. Although possibly slightly suboptimal compared to a dedicated workstation, this pragmatic approach was chosen in order to allow this study to take place outside the working environment. We don’t believe that this significantly altered the outcome of the study, as we were able to compare nodules detected by the study radiologists with clinical reports (and indeed found more nodules than initially reported).”

12. The concept of “center” of the nodule and adjudication of TP or FP with this concept is confusing. Please clarify and revise this part to make it easier to understand.

We thank the reviewer for this question and believe that revision of the manuscript is not necessary because this is a standard method. The CAD software should position and prompt within the center of any nodule, and we wished to find out of this prompt aligned with the mark-up of the two radiologists. We then applied the Dice coefficient method to detect discrepancies in the segmentation between the software and the various observers. This method is described in the methods section, and is a common way of demonstrating the level of overlap between the different segmentations in these types of studies.

13. The authors mentioned that group 1 consisted of 178 CT scans being free from nodules. How was sensitivity calculated from group 1 without nodules?

We can be very brief answering this question: sensitivity is calculated on lesion level, not on scan level. We hope the reviewer will be satisfied with this answer.

14. Is it possible to statistically compare diagnostic performance between the CAD alone and radiologists with or without CAD?

Yes, this was the set-up of our study as described in the re-written part of the “Image Annotation” chapter of the Materials and Methods section. We have rewritten this part as per suggestion of the reviewer (comment #9). We hope we made this section more clear and thank the reviewer for notifying the opaqueness of this part.

**Reviewer #2:** I want to thank the authors for the additional effort that went into this manuscript, and for clarifying some of my comments.
I am however not satisfied with how the authors addressed my main points of criticism.
The reference standard is set by using the tested CAD system at a high sensitivity (and high FP rate) setting, and the same readers that set the reference standard are used for testing whether CAD helps them. This introduces a bias, even if there is a third experienced radiologist as an adjudicator. The authors have not addressed my suggestions for compensating for this. Another alternative solution that I did not mention before would be to take the full set of marks found by the two readers during the reading session (50% aided, 50% unaided) and have a new panel of 3 radiologists review this consolidated set of marks and set the reference standard. Note that this reference panel should then be blinded as to whether the mark that they are presented with was detected by a reader only, or by the reader following a CAD prompt.

Answer to Reviewer #2: We would like to thank Reviewer #2 for her/his critical appraisal of the manuscript. In this and the previous review-round, Reviewer #2 suggests to perform additional studies with more readers to confirm the results described in this manuscript. However, we choose not to invest in further analyses of a study that already has been completed, but to invest in additional research questions instead. There are more studies with the same software ongoing and published, please search for “Aidence” in PubMed.

In this manuscript, we describe the validation of software, that was initially developed with lung cancer screening data, in “everyday clinical use”. In this routine clinical practice we aim for high detection rates of nodules because of their potential malignancy. Therefore, we apply a CAD system with high sensitivity. Radiologists were allowed to accept or refute marks indicating potential nodules. This is a commonly applied methodology, as it allows the detection of nodules otherwise missed, while the radiologist can discount “over call”. This is how a system works in clinical practice when aiming for high detection rates: high sensitivity at a cost of decreased specificity. Examples include mammography screening and also screening tests such as cervical smear testing and occult blood testing for colon cancer.

We do acknowledge the concern of reviewer #2 and have addressed this as a limitation of our study in the discussion. At the time of the first clinical validation of our novel software, time and budget were limited. However, we believe that the study described in this manuscript is fair and significant, despite the constraints we had to face when conducting it.

**Reviewer #2:** Finally, the claim that the software helps radiologists is with the current data only partly supported when the CAD software is set at this high sensitivity threshold. Using the presented data, we cannot make conclusions as to whether the CAD software would still help radiologist when it is used at a different, more clinically acceptable operating point of 1 or 2FPs on average per scan.

Answer to Reviewer #2: We thank the reviewer again and we respectfully disagree. What the reviewer claims as clinically acceptable is actually very close to what we were seeing in the aided reads: an average FP rate of 0.11 (radiologists without CAD) and 0.16 (radiologists with CAD) per CT scan. This merely reinforces what we stated above: we require high sensitivity settings in order not to miss actionable nodules. We have addressed the limitations of the study in our discussion and we believe that we validated the point of our retrospective study: showing that this software could help radiologists in their daily clinical practice to not miss pulmonary nodules in patients.
