# Supplementary material for: Validation of a deep learning computer aided system for CT based lung nodule detection, classification, and growth rate estimation in a routine clinical population
Source: PLoS One. 2022 May 5;17(5):e0266799. doi: 10.1371/journal.pone.0266799 (PMC9070877; doi:10.1371/journal.pone.0266799)
Supplement: Attachment — Submitted filename: Response to Reviewers.docx [file pone.0266799.s004.docx]

# **Rebuttal Letter, 3^rd^ response to reviewers**

9 February 2022

**Reviewer #1:** First of all, thank you for your submitting your manuscript with appropriate revision. I think your revision was very appropriate and will make future readers’ concerns alleviated.

**Corresponding author**: thank you for your contributions and critical appraisal of our manuscript, reviewer #1.

**Reviewer #2**: I want to thank the authors for their responses.

The rebuttal letter reads that the authors respectfully disagree with my statement "we cannot make conclusions as to whether the CAD software would still help radiologist when it is used at a different, more clinically acceptable operating point of 1 or 2FPs on average per scan." Based on the response in the letter, it is my understanding that the authors disagree that 1 or 2 FPS on average per scan is a more clinically acceptable operating point? This surprises me because the authors write in the discussion: "A more acceptable average FP rate would be between 1 and 2 with corresponding sensitivity range (82.3% - 89.0%), outperforming thoracic radiologists with and without using CAD."
So, I think the authors and me actually agree on what is a clinically acceptable setting, so I do not fully understand the response. My main point is that this study shows the CAD system set to operate at 7 FPs per scan helps increasing sensitivity of radiologists, but has no direct evidence what the added performance would be when the CAD is set to operate at 1 or 2 FPs per scan. Do the authors disagree with that?

**Corresponding author**: first of all, we (the corresponding author and the radiologists involved in the study) would like to thank reviewer #2 for the discussion and critical comments regarding our manuscript. In the end, this is what creates progress in science. Regarding your point above, we do not disagree. The primary purpose of our study was to validate the detection, segmentation, classification, and growth assessment of our software device in clinical practice. Not the detection, nor its impact on reader performance. The operating point was therefore set to an extreme level to make sure that the ground truth was as complete as possible. We agree with reviewer #2 that we would never use an OP of 7 FP per scan in clinical practice. We therefore will adopt the manuscript as suggested.

**Reviewer #2**: The authors have decided to not do additional analysis and I respect that. I understand that priorities shift over time and that time and resources are limited. The concerns that I described are to a certain extent covered in the Discussion section.

**Corresponding author**: we would like to thank reviewer #2 for her/his understanding of shifting our priorities. We have invested our resources in an FDA study for 510(k) approval, of which we will publish the results as well.

**Reviewer #2**: If this paper is to be published in its current form, I think it is necessary to at least perform the following changes:

- As discussed in the first paragraph in the Discussion, the threshold of 0.1 was used in this study for the CAD, corresponding to a sensitivity of 95% and a false-positive rate of 7 on this dataset.
Since the CAD is used by the readers at this operating point, I think the result section of the abstract should report this performance, and not the performance at another operating point. Especially because the next sentences are about radiologist performance with or without CAD. That is confusing and misleading, in my opinion.
So, I think the first sentence of the Result section of the Abstract should read:
"After analysis of 470 pulmonary nodules, the sensitivity of CAD as a stand-alone test for detecting nodules was 95% with an average FP rate of 7 per CT scan at the operating point used in this study."

**Corresponding author**: in concordance with the points you raised above and in previous review rounds, we agree with you. Therefore, we have decided to completely remove the sensitivity and FP numbers from the abstract (please see the revised manuscript with track changes). After all, the purpose of the study was to validate an algorithm and therefore the radiologist performance with or without CAD is the main point of the study.

**Reviewer #2**: The discussion section reads "These findings compare favorably with several other software tools (21,23)." I think this statement is not fair and not proven by the current results and with the current reference standard. Please remove this part.

**Corresponding author**: yes, we agree with this suggestion and have removed the sentence from the manuscript.
