# Supplementary material for: Validation of a deep learning computer aided system for CT based lung nodule detection, classification, and growth rate estimation in a routine clinical population
Source: PLoS One. 2022 May 5;17(5):e0266799. doi: 10.1371/journal.pone.0266799 (PMC9070877; doi:10.1371/journal.pone.0266799)
Supplement: Attachment — Submitted filename: Response to Reviewers.docx [file pone.0266799.s005.docx]

**Rebuttal Letter, 4^th^ response to reviewers**

25 March 2022

**Reviewer**: 1. line 34-35: "the readings for radiologists without CAD and radiologist with CAD, were 71.9% ... and 80.3%.." --> The meaning of each number is not clear by this sentence, and thus should be properly revised.

**Corresponding author**: thank you for highlighting this error, the numbers indicate sensitivity and we have added this to the text.

**Reviewer**: 2. line 45-47: "Results suggest this software could assist chest radiologists in pulmonary nodule detection and management within their routine clinical practice."--> I think this statement cannot be supported by the results, so I recommend to remove this part or the authors might want to revise as "The deep Learning software has the potential to assist radiologists in the tasks of pulmonary nodule detection and management on routine chest CT, which was written in the Discussion section by the authors.

**Corresponding author**: thank you for your suggestion, we have adapted the text accordingly, see “track changes”.

**Reviewer**: 3. line 82-84: "Lung cancer remains the third most prevalent cancer worldwide, is both rising in incidence (1), and maintains high mortality rates with around 1.7 million global deaths annually."
--> Reference #1 is too much out-of-date. The authors need to update the reference.

**Corresponding author**: the reviewer is right, new data indicates that lung cancer mortality rates are even 1.8 million global deaths. We have edited the text and the reference accordingly.

**Reviewer**: 4. line 290-292: "the results of our study are of similar sensitivity and accuracy to that initial cohort, (87% at 1 FP/scan) confirming broader use is feasible."--> This part needs a reference.

**Corresponding author**: we have added the reference.

**Reviewer**: 5. line 337-340: "The Deep Learning model for nodule detection was trained on data from a lung cancer screening cohort but this study shows that it is effective in a general, “real life” clinical setting where it improves the sensitivity of detection of actionable nodules by thoracic radiologists." --> this statement went too far and actually was not supported by the results. So the authors might want to remove this part.

**Corresponding author**: we have altered the original sentences, see track changes, and weakened our initial conclusion. We expect that these new sentences will be in line with what the reviewer suggests.
